# The influence of mutually beneficial and self-beneficial situations on the lying behavior and cognition of Chinese 4- to 5-year-old children

**DOI:** 10.3389/fpsyg.2025.1513033

**Published:** 2025-01-30

**Authors:** Dan Kang, Yingjie Zhang, Xiwu Xu, Jiajia Li

**Affiliations:** ^1^Key Laboratory of Cognition and Human Behavior in Hunan Province, School of Educational Science, Hunan Normal University, Changsha, China; ^2^Faculty of Education, University of Macau, Taipa, Macao SAR, China

**Keywords:** mutually beneficial, self-beneficial, lying behavior, lying cognition, young children

## Abstract

**Background:**

Children’s moral development is influenced by their sociocultural context. However, relatively few studies have investigated whether the sociocultural context affects children’s lying behavior and cognition and the relation between them.

**Methods:**

The present study was designed to examine this question in two experiments by posing two moral dilemmas: lying is good for mutually beneficial (honesty conflicts with mutual interests) and lying is good for self-beneficial (honesty conflicts with self-interests). Experiment 1 used the “hide-and-seek” game to investigate the lying behavior of 96 Chinese 4- to 5-year-old children. Experiment 2 used two videos to investigate lying cognition (conceptualization of lying, moral assessment of lying, and prediction of lying) with the same group of children.

**Results:**

In Experiment 1, children lied more in mutually beneficial situations than in self-beneficial situations. Experiment 2 revealed that, compared with self-beneficial situations, children in mutually beneficial situations were more likely to judge untrue statements as truth, to evaluate lying positively, and to predict that they would lie. Further, in mutually beneficial situations, children’s predicted and actual lying behaviors are significantly positively correlated.

**Conclusion:**

These findings support the folk model and highlight the influence of sociocultural factors on lying in Chinese 4- to 5-year-old children. They offer meaningful insights into the development of early moral understanding and behavior.

## Introduction

Affection for the collective and honesty are both moral virtues actively fostered in human society. However, people often encounter situations in their daily lives in which honesty conflicts with the common interests of the collective. For example, whether to lie to their furious boss on behalf of their colleagues for the sake of their department’s reputation at work or help their teammates cover up fouls during a competition to ensure their team remains in the lead. Solving this kind of problem is difficult for many people, let alone young children. Hence, how do young children cope with value conflicts in life? Do they uphold the principle of honesty at the expense of mutual interests, or do they disobey the principle to defend them? Exploring these questions is critical for understanding the development of children’s lying behavior and lying cognition and improving early childhood moral education practices. Therefore, in this study, we created two moral dilemmas in which mutual or self-interests conflict with the principle of honesty to observe how collective value orientation, which aims to maximize mutually beneficials, influences lying in children aged 4–5 years.

### Lying for mutual or self-interests

Lies can be classified into two types based on the motivation behind them: lies told for mutual interests and lies told for self-interest. Lies told primarily to benefit both oneself and others are called “collective lies” or “blue lies” (Barnes, 1994; Klockars, 1984), while those primarily used to protect one’s own interests are referred to as “selfish lies” (Cheung et al., 2016). Selfish lies are generally not respected; however, collective lies are commonly accepted within specific social contexts (Barnes, 1994; Bok, 1978; Fu et al., 2007). This moral flexibility may stem from lying situations. According to the folk model (Holland and Quinn, 1987), cultural factors or social interaction motives are more likely to influence an individual’s understanding of lying in social situations. Therefore, in some social contexts dominated by the principle of maximizing mutual interests, the focus of verbal statements is not on the accuracy of information or whether truthful information hurts others’ feelings but rather on whether the information helps achieve common goals and interests. Consequently, in such situations, a false statement that benefits the common good is often morally forgiven (Mojdehi et al., 2020a).

Research on collective lies upon different cultural contexts supports the folk model. For example, Mojdehi et al. (2020a) found that Canadian children rated collective lies negatively, which is consistent with the value systems of their own culture. Even though Canada is known as a multicultural society, it is categorized as individualist, which values individual interests over groups (Oyserman et al., 2002). These individualistic values are clearly mirrored in Canadian children’s moral evaluations of collective lies. In contrast, Fu et al. (2007) showed that, relative to Canadians, 7-, 9-, and 11-year-old Chinese children place higher value on collective lies than selfish lies. Furthermore, as age increased, Chinese children increasingly favored lying to benefit the collective over an individual. The importance of collectivism and one’s dedication to a group of people or one’s family is a strong belief in Chinese culture, even though it originates not only from religion (Fatehi et al., 2020). For instance, an individual is expected to sacrifice his personal desires in order to serve a large group or his family as a whole. Thus, in a society with a collective value orientation that aims to maximize mutually beneficials, children tend to appreciate collective lies even if they contradict the truth. These findings suggest that cross-cultural differences in emphasis on collectives versus individuals affect children’s choices and moral judgments about collective lies and selfish lies.

### Lying for mutual or self-interest among preschool-age children

Since stressing collectivism is the main belief in some countries (Périard and Liu, 2020; Mojdehi et al., 2020b), the way of interaction in their kindergartens has a strong collectivistic orientation. For example, in Chinese kindergarten, most of the day, the children fulfilled activities in a large group, like singing, drawing, having a lesson, moving around in the playing area, etc. Every single member is supposed to comply with the group in order to enable the group’s benefit. For instance, when children were lining up on orientation lines in the classroom, every single child had to do this correctly in order to be able to leave the classroom as a group (Périard and Liu, 2020). Thus, preschool-age children who receive collectivist early education could easily adopt the collective concept, which has been reflected in their social behavior and judgment, such as helping (Orlick et al., 1990), sharing (Rao and Stewart, 1999), and prosocial lying (Mojdehi et al., 2020b). To date, however, little is known regarding whether preschool-age children from collectivist cultural backgrounds are willing to tell collective lies, a prosocial behavior contrary to the principle of honesty, and how they interpret such behavior.

Piaget (1932) proposed that children’s sensitivity to prosocial versus antisocial moral intentions as well as to other facets of intentionality does not emerge until around age 7–8. This is consistent with previous research, showing that Chinese 7-year-old children have a tendency to lie for mutual interests and reach a near-adult level by age 11 (Fu et al., 2007; Lau et al., 2013). However, evidence indicates that even children as young as 4 begin to consider the motives behind lies. For instance, Peterson and Siegal (2002) found 44% of 4-year-olds discriminated on the basis of intention by rating deliberate lies as morally worse than unintentional falsehoods, indicating an early sensitivity to the intentions behind dishonest behavior. In the Chinese cultural context, which places high value on group harmony and collective interests, this early awareness of lying intention may promote a moral understanding of collective lies at a younger age. While most studies have focused on older children, further investigation into preschool-aged Chinese children’s responses to collective lies could provide valuable insights into how cultural norms shape early moral reasoning. Therefore, the current study created two moral dilemmas to verify whether the principle of pursuing the maximization of mutual interests affects preschool-age children’s lying behavior and cognition and the relationship between them.

### Separation of knowledge and action in lying in preschool-age children

Lying cognition concerns children’s conceptualization of lying and the moral assessment and prediction of their lying behavior (Zhang et al., 2007). Lying behavior is defined as an act in which someone intentionally fabricates events or distorts facts to mislead and convince others (Lee, 2000). It is critical to establish a connection between children’s lying cognition and behavior because ensuring that children not only know what is morally right or wrong but also act accordingly is the ultimate goal of socialization. However, several studies found a phenomenon of “separation of knowledge and action” in young children’s lying (Talwar and Lee, 2008; Talwar et al., 2002, 2004). For example, Talwar et al. (2002) found that although children aged 3-to-7 years were able to accurately understand the meaning of lying and identify that lying should be criticized, they still lied to conceal their own transgression during an experiment requiring them to resist temptation, showing inconsistencies between knowledge and behavior. This means that there appears to be a weaker association between lying cognition and behavior in young children.

The separation of knowledge and action in lying supports social cognitive theory (Bandura, 1986; Batson et al., 1997), in that no correlation exists between children’s moral cognition and behavior because their speech and behavior are two separate aspects. However, social cognitive theory mentioned that “although the judgment of good or bad moral behavior has nothing to do with moral behavior itself, situational factors such as the social learning process, the situation, the level of psychological development, and unconscious behavioral tendencies may be the factors that determine whether moral behavior occurs.” Damon (1999) further emphasized that children’s moral development is shaped by social interactions and cultural contexts, which encourage behaviors that support collective goals. That is, the relationship between lying behavior and cognition in children may vary across contexts. Fu et al. (2008) revealed that when Chinese children’s moral evaluation scores of lying for self, lying for a collective, truth-telling for self, and truth-telling for a collective were entered into the regression model, a significant correlation was only found between children’s actual collective lie-telling behavior and their moral choice and judgment scores for lying for the collective. It suggested that in some culture where collectivist values emphasize group harmony and mutual benefit, the separation between lying behavior and moral cognition may be less pronounced, particularly in contexts of lying for collective. However, since existing research has mostly focused on school-age children, whether a phenomenon of separation of knowledge and action exists with respect to preschool-age children’s collective lies remains unclear.

### The current study

Based on the folk model (Holland and Quinn, 1987) and the work of Tsoi et al. (2021), the current study investigated the impact of situations on Chinese preschool-age children’s lying behavior and cognition and the association between them. To achieve our aims, we conducted two experiments in which children were required to respond to two moral situations: lying for mutual interests (the principle of honesty conflicts with mutual interests) and lying for self-interest (the principle of honesty conflicts with self-interests). In Experiment 1, children’s lying behaviors were investigated in two situations. Then, in Experiment 2, the same group of children’s lying cognition (i.e., conceptual understanding, moral evaluation, and behavior prediction) was tested in two situations.

## Experiment 1

In this experiment, we aimed to examine the effect of situations on children’s lying behavior. In a situation of mutually beneficial or self-beneficial, children aged 4–5 played a “hide and seek” game. The age range was chosen because prior studies found that 3-year-old children had a clear floor effect in lying tasks while 6-year-old children exhibited a ceiling effect (Wellman et al., 2001). Thus, similar situational differences were most likely to be seen in 4- and 5-year-olds. According to the folk model (Holland and Quinn, 1987), children will lie more often in situations of mutually beneficial if they are influenced by the principle of mutual interest first.

### Methods

#### Participants

A total of 100 participants were recruited in this study, and four of them were excluded due to: the participant not wanting to continue (1), the participant not understanding the rules (2), and the participant being too shy to respond (1). The final sample consisted of 96 participants (4-year-olds: *n* = 48, mean age = 4.55, range = 4.24–4.92; 5-year-olds: *n* = 48, mean age = 5.56, range = 5.17–5.89). Among the final sample, there were 48 boys (50%) and 48 girls (50%), with an equal gender distribution across both age groups. A sensitivity analysis conducted in G*Power 3.1 (Faul et al., 2009) showed that the final sample of 96 provided 95% power (1 – *β* = 0.95) in detecting a correlation with a medium effect size of *ρ* = 0.33 (two-tailed). All the children were recruited from a kindergarten in the middle of mainland China, and their families came from a variety of social and economic backgrounds. All study procedures were approved by the local institutional review board of Hunan Normal University, and informed consent was collected from the parents.

#### Materials

Two graduate students majoring in preschool education conducted one-on-one experiments with children in a quiet room in a kindergarten. Two boxes of exactly the same size (10 cm × 10 cm × 10 cm) were provided. One box was red and the other was green. Before the study began, the children were offered 10 similar items for each type of reward and asked to choose which they liked best. The reward with the most votes was used for the experiment. The goal of the game was to obtain as many rewards as possible. Another child, matched gender and age, acted as the child’s partner during the game.

#### Procedure

All the children were asked to complete two 15-min games of “hide and seek” in random order, one in the self-interest situation and one in the mutually beneficial situation. In the self-interest situation, only one person could win the reward at a time. Firstly, children were asked to hide a reward in either the red or green box while their partners turned and closed their eyes. After the reward was hidden, the partner opened their eyes and asked the child where it was. Then, the child was asked to respond (e.g., by pointing to either the red or green box). Finally, the partner needed to guess the location of the reward based on the child’s response; however, they could only follow the child’s suggestion and made passive guesses. If the child pointed to the correct box, the partner could find and keep the reward; if the child pointed to the empty box, the partner would not find any reward, and the child could keep it. In the mutually beneficial situation, children were asked to hide two rewards in the same box, and two people could win rewards at the same time. The partner also could only choose the box indicated by the child. If the partner guessed correctly, neither person would receive a reward, but if the partner guessed incorrectly, both the child and their partner would receive a reward. The relevant observation was whether the child misled their partner by providing incorrect answers. If a child gave the correct answer, it was regarded as telling the truth. Otherwise, it was regarded as a lie. Four game rounds were conducted. One score was given for each lie; thus, total scores ranged from 0 to 4.

The children responded to two comprehension check questions: (a) if your partner guesses correctly, who keeps the reward, and (b) if your partner guesses incorrectly, who keeps the reward? If a question was answered incorrectly, the rules were explained to the child again. Children were excluded from the sample if they did not demonstrate a basic understanding of the rules after they were explained three times (this affected two participants). After the game, we asked children a question about the last trial to check for differences in their short-term memory: “Which cup did your partner point to last?”.

### Results

#### Preliminary analyses

Table 1 provides the basic descriptive and correlational statistics of the critical and demographic variables. The number of children’s lying times revealed that 4-to-5-year-old Chinese children not only would lie for their own benefit (selfish lie), but also are willing to lie prosocially to protect the mutually beneficial of themselves and others (collective lie). This may suggest that children have developed some of the skills necessary for lying, i.e., theory of mind (Sai et al., 2021) and executive function (O'Connor et al., 2020). In addition, the number of more than half of children who tell collective lies preliminarily suggests that even children as young as 4 and 5 years old have an understanding of social norms and the emotions of others (Demedardi et al., 2021; Fu et al., 2016). To examine whether children’s lying behavior diverged significantly from the chance level (50%), we conducted one-sample *t*-tests for each situation and age group. Results showed that for both the mutually beneficial (collective) situation and the self-beneficial situation, both 4-year-olds [mutually beneficial: *t*(47) = 4.32, *p* < 0.001; self-beneficial: *t*(47) = 2.15, *p* = 0.037] and 5-year-olds [mutually beneficial: *t*(47) = 5.14, *p* < 0.001; self-beneficial: *t*(47) = 3.68, *p* = 0.001] lied significantly more than would be expected by chance. The results of correlation analysis showed that children’s lying times in both situations were not related to gender but were related to age. Therefore, age will be included in the subsequent data analysis.

**Table 1:** The difference in the children’s lying times in different situations and ages in Experiment 1 (*M* ± SD).

	Situation		*F*	Age		*F*
	Self-beneficial	Mutually beneficial		Four-year-old	Five-year-old	
Lying times	2.04 (1.43)	2.67(0.58)	8.79^**^	2.60 (1.30)	2.54 (1.46)	0.28

^*^p < 0.05, ^**^p < 0.01, ^***^p < 0.001.

We checked the assumptions for conducting the analysis of variance (ANOVA). The scores of each outcome variable were consistent with the normality principle in our study since all the values of skewness and kurtosis were between-1 and 1 (as suggested by Tabachnick et al., 2013). Non-significant Mahalanobis distances (*p* > 0.050) showed no multivariate outliers. Both Box’s Test of Equality of Covariance Matrices (*p* > 0.001) and Levene’s Test of Equality of Error Variance were not significant, indicating that the data followed the assumptions of homogeneity of covariance and variance required for ANOVA. Based on these results, an ANOVA was subsequently performed. The *p*-values reported in the following analyses have been Bonferroni corrected.

#### Main analyses

In order to examine which factors affect children’s lying behavior, we conducted a univariate analysis of variance (ANOVA), with situation and age as independent variables and lying times as dependent variables. The results are shown in Table 1. This ANOVA revealed that the main effect was significant in two situations (i.e., mutually beneficial or self-beneficial), *F*(1,95) = 8.79, *p* = 0.001, *η^2^_p_* = 0.100. Children lie more often in the situation of mutually beneficial (*M* = 2.67, SD = 0.58) than in the situation of self-beneficial (*M* = 2.04, SD = 1.43). Although the correlation analysis showed that children’s lying times in both situations were related to age, the main effect of age, *F*(1,95) = 0.28, *p* = 0.602, *η^2^_p_* = 0.006, was not significant. There was no significant difference in the number of lies between 4-year-old (*M* = 2.60, SD = 1.30) and 5-year-old children (*M* = 2.54, SD = 1.46). In addition, the interaction between age and situation, *F*(1,95) = 1.67, *p* = 0.20, *η^2^_p_* = 0.020, was also not significant.

### Discussion

As expected, the situation had a significant impact on Chinese preschool children’s lying behavior. Children were more willing to tell a collective lie than a selfish lie. When honesty conflicted with mutual interests, children prioritized the latter. The findings indicate that the lying behavior of Chinese 4-to-5-year-old children is significantly affected by the principle of pursuing the maximization of mutual interests. This aligns with previous evidence that primary school children in collectivist cultures engage in more lying behaviors when the aim is to help a group (Fu et al., 2007; Lau et al., 2013; Lim et al., 2020), suggesting that children’s moral behavior are probably influences d by cultural traits.

## Experiment 2

The results of Experiment 1 showed that, when children decide whether to lie in a dilemma situation, they are more likely to lie for the mutually beneficial of themselves and a partner. It is worth noting that there may be a separation between children’s moral behavior and cognition. However, previous studies have found that children tend to show more consistent cognition and behavior when lying for mutually beneficial (Fu et al., 2008). For example, children lied during an experiment requiring them to resist temptation, but not before they made it clear that they knew the meaning of lying and believed it was not good, showing inconsistencies between knowledge and behavior (Talwar et al., 2002). However, it is not clear how younger children (for example, 4–5 years old) recognize and evaluate collective lies and whether the relation between their perceptions and actual behavior varies depending on the purpose of the lie. Therefore, we tried to answer these questions in Experiment 2.

### Method

#### Participants

Participants were the same group of children from Study 1. A total of 96 children completed this study. A sensitivity analysis conducted in G*Power 3.1 (Faul et al., 2009) showed that our sample provided 95% power (1 – *β* = 0.95) in detecting a correlation with a small effect size of *ρ* = 0.10 (two-tailed). Two months elapsed between Experiment 1 and Experiment 2. By asking questions, we ensured that the children had forgotten the content of Study 1 and avoided the practice effect.

#### Materials

The materials used in Experiment 2 were two videos created in a university laboratory. Each video used the same boxes and rewards as in Study 1; however, the players were two cute puppets of equal size (15 cm × 25 cm) and appearance: Puppet A (a rabbit in a blue hat) and Puppet B (a rabbit in a yellow hat). Puppet A used a little girl’s voice, Puppet B used a little boy’s voice, and the game host used an adult male voice. The two videos corresponded to two situations (mutually beneficial and self-beneficial). In the self-beneficial situation, Puppet A hid a reward in one of the two boxes (red or green), while Puppet B closed his eyes. Puppet A responded (e.g., by pointing to either the red or green box) when Puppet B opened his eyes and asked Puppet A where the reward was. Puppet B would then guess the location of the reward based solely on Puppet A’s response. In this condition, if puppet B guessed correctly, Puppet B received the reward; however, if Puppet B guessed incorrectly, the reward went to Puppet A. In the mutually beneficial situation, Puppet A hid two rewards, with both going into the same box (red or green). In this condition, if Puppet B guessed correctly, neither puppet received a reward; however, if Puppet B guessed incorrectly, Puppet A and Puppet B each received a reward. In both videos, Puppet A hid the reward and then told Puppet B that it was in the other box, while Puppet B “believed” Puppet A every time and always guessed incorrectly. In Video 1, Puppet A lied and received the reward together with Puppet B; in Video 2, Puppet A lied and received the reward alone. The videos ranged from 80 to 100 s in length.

#### Procedure

An adult female experimenter conducted the experiments alone with the children in Chinese in a quiet room in a kindergarten. All the children watched two videos on a 10.2-inch iPad. Each child took 20 min to complete the experiment. To control for the order effect of the videos, half of the participants watched the videos in random order and the other half in reverse order. After watching each video, children were asked to answer three control questions to assess their understanding: (a) if Puppet B guessed correctly, who would receive the reward; (b) if Puppet B guessed incorrectly, who would receive the reward; and (c) in which box did Puppet A hide the reward? If a question was not answered correctly, the rule was explained to the child again. After receiving the explanation three times, children who did not demonstrate a basic understanding of the video were excluded from the study (all children successfully answered the control questions correctly). The children were also asked three questions about lying cognition: concept understanding, moral evaluation, and behavior prediction. To avoid order effects, children in both the sequential and reverse groups were randomly divided into two groups. One group answered questions in order A (Q1–Q2–Q3), and the other answered questions in order B (Q3–Q2–Q1).

Q1. Understanding of the concept of lying: “Did Puppet A lie?” Whether the child answered yes or no, the next question was, “Did Puppet A tell the truth?” These two questions reflected the child’s understanding of the concept of lying. Only when both questions were answered correctly can children understand that lying and telling the truth are recorded as 1.

Q2. Moral evaluation of lying behavior: “Is it good or bad for Puppet A to say so?” This question was scored as 1 for a positive evaluation and 0 for a negative evaluation. Then, a seven-point evaluation card scale [26] was used to ask children to evaluate the degree of “good or bad.” Children were told that red stars (★) represented good and black crosses (×) represent bad: ★★★ indicated “very good” (recorded as 3), ★★ indicated “relatively good” (recorded as 2), ★ indicated” a little good” (recorded as 1), ××× indicated “very bad” (recorded as-3), ×× indicated “relatively bad” (score-2), × indicated “a little bad” (recorded as-1), and ○ indicated “neither good nor bad” (recorded as 0). The images on the cards ensured that children of all ages could understand it.

Q3. Prediction of lying or telling the truth: “If you were Puppet A, which box would you tell Puppet B the reward was in?” Providing an incorrect answer indicated that the child predicted that they would lie, which was recorded as 1 point; a correct answer indicated that the child predicted that they would tell the truth, which was recorded as 0.

### Results

#### Preliminary analyses

Table 2 provides the basic descriptive and correlational statistics of all the variables. The correlational analysis revealed that the results of all aspects of children’s lying cognition are consistent with each other. Children who are more likely to identify a statement as a lie tend to evaluate such statements more negatively, and they are less likely to predict that they will say such statements themselves. Similar to lying behavior, children’s perception of lying is only related to their age, rather than their gender. Thus, age will be considered as one of the predictors in the main analysis.

**Table 2:** The difference in the children’s lying cognition in different situations and ages in Experiment 2 (*M* ± SD).

	Situation		*F*	Age		*F*
	Self-beneficial	Mutually beneficial		Four-year-old	Five-year-old	
Conceptual understanding	4.29 (2.61)	2.77 (2.55)	15.63 ^***^	6.30 (4.19)	7.89 (3.97)	6.78^*^
Moral evaluation	−1.02(0.87)	2.66 (1.45)	86.64 ^***^	1.23 (11.79)	0.78 (12.74)	49.59^*^
Behavioral prediction	3.90 (1.35)	4.29 (1.44)	32.45 ^***^	7.44 (1.88)	9.00 (2.21)	4.0^*^

^*^p < 0.05, ^**^p < 0.01, and ^***^p < 0.001.

The assumptions for conducting the ANOVA were also checked, and the results indicated that the data followed the assumptions of homogeneity of covariance and variance required for an ANOVA. The *p*-values reported in the following analyses have been Bonferroni corrected.

#### Main analyses

##### Effect of situation on children’s lying cognition

The results of the 2 (situations: mutually beneficial or self-beneficial) × 2 (ages: 4 or 5) repeated measures ANOVA showed that the main effects of situation on children’s conceptual understanding [*F*(1,95) = 15.63, *p* = 0.0008, *η^2^_p_* = 0.14], moral evaluation [*F*(1,95) = 86.64, *p* = 0.0005, *η^2^_p_* = 0.48], and behavioral prediction [*F*(1,95) = 86.64, *p* = 0.0005, *η^2^_p_* = 0.48] (as shown in Table 2). If the purpose of an untrue statement was to obtain a mutually beneficial outcome, children perceived it more as truth, were more willing to provide positive comments encouraging this behavior, and were more likely to think they would do the same. The results also showed the main effects of ages on children’s conceptual understanding [*F*(1,95) = 6.78, *p* = 0.015, *η^2^_p_* = 0.07], moral evaluation [*F*(1,95) = 49.59, *p* = 0.021, *η^2^_p_* = 0.04], and behavioral prediction [*F*(1,95) = 4.0, *p* = 0.023, *η^2^_p_* = 0.06] were significant. Compared with 4-year-old children, 5-year-old children can more precisely identify statements that are inconsistent with the truth and rely more on veracity to make moral judgments. And 5-year-old children were significantly less likely than 4-year-old to believe they would lie, both collectively and selfishly. No significant interaction was found between age and situation.

##### Correlation between children’s lying behavior and cognition

A correlation analysis was conducted to test whether there was a separation between children’s lying behavior and cognition. As shown in Table 3, no significant correlations were found between lying behavior and conceptual understanding and moral evaluation, whether lying was beneficial to mutual interests or self-interest; however, only when lying was beneficial to the collective was actual lying behavior significantly and positively correlated with predicted lying behavior, *r* = 0.37, *df* = 94, *p* = 0.0001. For both 4-year-old children, *r* = 0.41, *df* = 94, *p* = 0.002, or 5-year-old children, *r* = 0.42, *df* = 94, *p* = 0.003, the correlation coefficient between actual lying behavior and predicted lying behavior was significant. Thus, in the mutually beneficial situation both 4-and 5-year-old children who predicted that they would lie did so in the actual situation.

**Table 3:** Correlation coefficient between children’s lying behavior and lying cognition.

Age	Self-beneficial			Mutually beneficial		
	Conceptual understanding	Moral evaluation	Behavioral prediction	Conceptual understanding	Moral evaluation	Behavioral prediction
4	0.14	−0.06	0.31	0.01	−0.07	0.41^**^
5	−0.22	0.18	0.12	−0.18	0.15	0.42^**^
All	−0.004	0.08	0.19	−0.05	0.04	0.37^***^

^*^p < 0.05, ^**^p < 0.01, ^***^p < 0.001.

### Discussion

The results of Experiment 2 support our hypothesis that the situation has a significant impact on Chinese 4- to 5-year-old children’s lying cognition, which is consistent with previous studies (Ma et al., 2011; Mojdehi et al., 2020a; Xu et al., 2010). These findings reflect the influence of social values that seek to maximize mutual interests on Chinese preschool children’s moral understanding. Notably, unlike for lying behavior, we found a significant age difference for lying cognition. Compared to 4-year-old children, 5-year-old children were more likely to identify statements that were inconsistent with facts, made more negative moral evaluations of lying behavior, and had a lower likelihood of predicting that they would lie. These results are also in line with those of previous studies (Bussey, 2003; Heyman et al., 2010; Maas, 2008; Vendetti et al., 2018), which showed that the continuous development of children’s moral cognition with age may be related to their increasingly rich life experiences, the maturity of their psychological abilities, and the gradual improvement of their intellectual development (Zhao et al., 2021). In addition, the correlation analysis results partially support social learning theory (Bandura, 1986; Batson et al., 1997), indicating that how children evaluate lying and how they choose their actual behavior are two independent aspects. Notably, Chinese preschool children’s actual and predicted lying behavior were not significantly correlated in the self-beneficial situation but were significantly positively correlated in the mutually beneficial situation. These findings show that, when lying is beneficial to both parties, the consistency between lying behavior and cognition is enhanced.

## General discussion

The current study examined how situations affect young children’s lying behavior and cognition and the relationship between them. We obtained three major findings. First, the situation had a significant impact on children’s lying behavior and cognition. Second, a significant difference was found based on age in children’s lying behavior but not their lying cognition. Third, while lying behavior had nothing to do with conceptual understanding or moral evaluation in either situation, a significant correlation was found between actual and predicted lying behavior in the mutually beneficial situation. These findings offer strong evidence for the folk model (Holland and Quinn, 1987), indicating that sociocultural factors significantly influence children’s lying behavior and cognition, which have important implications for understanding the nature of children’s moral development.

Consistent with previous research on Chinese school-age children (Fu et al., 2007, 2008), the current study found that lying behavior and cognition are influenced by the situation in Chinese preschool-age children. The folk model (Holland and Quinn, 1987), which emphasizes that “the idea of lying and its moral meaning are dependent on social customs,” is consistent with children’s moral decision-making in this study. In some cases (e.g., when social customs require lying to protect mutual interests) intentionally untrue statements are not labeled as lies, but are instead widely accepted and even encouraged (Keenan, 1976). Our results indicate that the collectivist value of pursuing the maximization of common interests in Chinese culture (Wang and Leichtman, 2000) is adopted by Chinese preschool-age children and helps guide their moral judgment. This contrasts with findings from individualistic cultural contexts, where children are more likely to emphasize personal interests and individual autonomy (Fu et al., 2007; Lau et al., 2013; Lim et al., 2020). For example, Lim et al. (2020) assessed the lying behavior of 77 Australian and 79 Singaporean children aged 6–12 and found that Australian children gave higher ratings to selfish lies and lower ratings to prosocial lies than their Singaporean peers. As shown in our study, Chinese children’s moral decision-making is likely to incorporate collectivist values, such as the need to protect mutual interests and maintain group harmony (Wang and Leichtman, 2000). The social learning theory (Bandura, 1986) helps explain these results by highlighting the role of both explicit and implicit social learning processes within the cultural environments in shaping moral cognition and behavior. Children in collectivist cultures may observe and internalize behaviors that emphasize group harmony and cooperation, leading to moral decisions that prioritize mutual benefit. In contrast, children in individualistic cultures may not develop the same emphasis on group harmony, instead internalizing norms that prioritize individual rights and personal honesty. The results of this study also support the view that children’s moral judgment rules are not a haphazard system, as they can derive priorities from different principles (e.g., the principle of maximizing mutual interests versus the principle of honesty). Young children may have a deep psychological framework that can assist them in developing interconnected moral norms. This interrelationship stems from the psychological representation of what makes someone moral. How this psychological representation changes with children’s development, life experiences, and age is an important issue that should be addressed in future research.

Mutually beneficial situations may foster children’s ability to empathize. Previous research has shown that when children are given prosocial incentives, their empathy ability and likelihood of lying improve significantly (Lim et al., 2020; Nagar et al., 2020). Mutually beneficial situations are equivalent to providing children with prosocial incentives that may encourage them to display empathy. In the mutually beneficial situation in this study, children and their partners were either not rewarded or rewarded together. This may have caused children to be less self-centered, think more about their partners’ interests, and consider their mutual interests as a whole. As a result, they were more willing to lie to avoid negative emotions from everyone, including themselves. However, in the self-beneficial situation, the children’s own interests collide with those of their peers. Consequently, the children may have been more concerned regarding whether they wanted a reward or what would happen if they lied, making their lying behavior and cognition self-centered. However, this study did not assess children’s empathy levels. Future studies could incorporate empathy and other personality traits to investigate the impact of individual personality trait distribution tendencies and their interactions on young children’s lying behavior and cognition.

In addition, two aspects of Chinese kindergarten education are likely to play a significant role in the influence of situations on children’s lying behavior and cognition (Lu and Gao, 2004; Qi and Tang, 2004). One is the daily collective activities of kindergartens. Zhang (2014) compared the one-day activities of kindergartens in China and the United States and found that in the United States, most of the daily activities of kindergartens are regional; urination, drinking water, and other life activities are carried out separately, while in China, collective activities accounted for the majority of daily activities, and many other activities were also performed as a collective, such as outdoor activities, physical exercise, and even games. Consequently, children must learn to coexist with team members and resolve conflicts between personal and mutual interests. Another is that educational content in kindergarten encourages children to increasingly integrate collective ideas. Compared to countries dominated by individualistic cultures, kindergartens in China are more likely to choose topics involving mutual interests, such as unity and cooperation, as educational content. Learning this content inevitably leads to an increasing preference for mutual interests in the development of moral behavior and cognition in Chinese children. Further, as children age, they become increasingly exposed to society’s cultural value systems. This may lead children to believe that lying is not always immoral and that they should consider factors other than authenticity when making moral judgments or decisions about lying (Fu et al., 2007, 2008). This development model is essentially consistent with Piaget’s moral development theory, which claimed that children may initially rely on simpler, more immediate rules, like honesty, but as their cognitive abilities and social experiences grow, they begin to recognize and integrate more complex principles, such as the importance of mutual interests (Piaget, 1932). Importantly, our findings suggest that this transition emerges as early as age 4–5 of this transition, rather than age 7–8 (Lau et al., 2013). This earlier development may be attributed to the sociocultural emphasis on collectivism, as Lim et al. (2020) found that mature awareness of the liar’s motivations develops significantly earlier in collectivist Singapore than in individualistic Australia. Future research could benefit from expanding the age range of participants and conducting cross-cultural comparisons to investigate how distinct sociocultural practices influence the transformation of moral reasoning.

In this study, 5-year-old children had significantly higher lying cognition than 4-year-old children; however, lying behavior did not differ by age. This indicates that, while children’s lying cognition improves significantly with age, their lying behavior does not. Lying cognition development may precede or be faster than lying behavior development. This could be attributed to two factors. First, this is linked to the one-sidedness of moral education (Huo et al., 2022; Korotaeva and Chugaeva, 2019; Xu, 2014). Currently, children’s moral education emphasizes knowledge over action, producing many “oral moralists,” which is likely to exacerbate the problem of inconsistency between words and deeds in children. Second, this could be linked to the intrinsic characteristics of children’s moral development (Dahl, 2019; Stapert and Smeekens, 2011). According to Piaget’s theory, moral cognition must precede moral behavior, as children need to develop an understanding of moral concepts before they can consistently act in alignment with them (Piaget, 1932). However, the transition from moral cognition to behavior is influenced by multiple factors, including cognitive development, emotional regulation, and personality traits (Talwar et al., 2002; Smith and Rizzo, 2017). As a result, this process is gradual and requires time for full integration of these components. Accordingly, moral behavior typically develops more slowly than moral cognition.

We also found no significant correlation between children’s lying behavior and their conceptual understanding and moral evaluation of lying. Previous research (London and Nunez, 2002; Talwar et al., 2002, 2004) has also shown that despite knowing something is a lie and that it is morally wrong, preschool children continue to lie when confronted with temptation. The phenomenon of “knowing and doing separation” could be caused by a lack of inhibitory control ability (Loke et al., 2011). Children must not only reject the allure of rewards but also suppress the dominant urge to lie when lying is the only way to obtain rewards. However, 4-to-5-year-old children have low inhibitory control (Geeraerts et al., 2020; Ghodrati et al., 2019; Livesey, 2000). To some extent, this is reflected in the age-related differences in lying behavior and cognition. Although 5-year-old children have higher levels of lying cognition, their poor inhibitory control may prevent or slow the transition from lying cognition to lying behavior. As a result, their lying behavior remains at the same level as that of 4-year-olds. Thus, there does not seem to be a clear one-to-one relationship between lying behavior and cognition, and a higher level of moral cognition does not always result in more positive moral behavior.

Notably, the current study found a strong positive link between children’s actual and predicted lying behaviors in mutually beneficial situations. Previous studies have also found that when children are given prosocial incentives (Popliger et al., 2011; Xu et al., 2010), their lying cognition becomes more congruent with their lying behavior. These findings reveal that children may conceal their true feelings and respond in ways that meet societal expectations. In social norms, lying for self-interest is often condemned because it contradicts ethical rules. Thus, although children may predict that they will tell the truth in self-beneficial situations (Caviola and Faulmüller, 2014), in reality, they often lie because of the enormous temptation of the reward. In contrast, in a mutually beneficial situation, lying for mutual interests is accepted and even encouraged by society (Lee, 2000; Zhao et al., 2021). Therefore, as children do not need to conceal their genuine thoughts, their predicted and actual lying behaviors are more consistent. Hiding their genuine thoughts to fulfill social expectations indicates that the theory of mind of children aged 4-to-5 years has matured to a certain level because they can recognize that their opinions differ from those of others and are able to transform others’ will into their own will to defend themselves.

Our findings have two implications for early moral education. First, teachers need to understand the social and cultural background of early moral education because it likely affects the development of children’s moral behavior and cognition. Second, teachers may notice that children do not always do what they say, especially when they are the only ones who benefit. To change this, children should be prompted to associate, recall, or reflect on their lying behavior and internalize the learned moral knowledge to achieve knowledge and practice unity.

### Limitations

The current study has three limitations. To begin with, this study relied on a small sample of 94 participants from a single kindergarten in mainland China, which may limit the generalizability of the findings. The results may not fully represent children from other regions of China or countries with similar collectivist values, such as Singapore or South Korea, due to cultural differences. Future research should include larger, more diverse samples across regions and cultures to strengthen and expand these conclusions. Second, each trial in this study only involved the gain or loss of one or two individuals, and the comparison may not be strong enough. Future research could create dilemma situations in which the principle of honesty conflicts with the interests at four levels: nation, kindergarten, class, group, and individual, in order to investigate children’s lying behavior and lying cognition under various degrees of interest conflict and psychological conflict. Third, we did not examine some factors that may moderate the influence of situations on children’s lying behavior and cognition, as well as their relationship, for example, empathy (Fu et al., 2018); theory of mind (Sai et al., 2020); and inhibitory control (Talwar et al., 2017; Williams et al., 2017). Finally, while the current study proves that children as young as 4 and 5 years old begin to lie for mutual interest, it is unclear when the socio-cultural context begins to affect children’s lying behavior and cognition. Figuring this out is crucial to further exploring the development of children’s lying behavior and cognition. Therefore, we encourage future research to expand the age range and include children under the age of 4 for additional investigation.

## Conclusion

We carried out two experiments to see if situations affect Chinese 4- and 5-year-old children’s lying behavior and cognition. The results indicate that (a) situations have a significant impact on children’s lying behavior and cognition and that (b) children’s lying behavior has nothing to do with conceptual understanding or moral evaluation, but when lies are told for mutual interest, the consistency between children’s actual lying behavior and their predictions of their own lying behavior is enhanced. Our findings support the folk model of lying and highlight the significance of socio-cultural factors in early moral education. Future research should look into the socio-cultural basis of lying, the long-term effects of collective ideas on children’s lying, and the relationship between lying and a wider range of values.

## Data Availability

The raw data supporting the conclusions of this article will be made available by the authors, without undue reservation.
